# Short-term complication rate following orthopedic surgery in a tertiary care center in Argentina

**DOI:** 10.1051/sicotj/2018027

**Published:** 2018-06-29

**Authors:** Gaston Camino Willhuber, Joaquin Stagnaro, Matias Petracchi, Agustin Donndorff, Daniel Godoy Monzon, Juan Astoul Bonorino, Danilo Taype Zamboni, Facundo Bilbao, Jose Albergo, Nicolas S. Piuzzi, Santiago Bongiovanni

**Affiliations:** 1 Hospital Italiano de Buenos Aires, Buenos Aires Argentina; 2 Hospital Italiano de San Justo “Agustin Rocca”, Buenos Aires Argentina; 3 Department of Orthopaedic Surgery & Biomedical Engineering, Cleveland Clinic, Cleveland USA; 4 Instituto Universitario del Hospital Italiano de Buenos Aires, Buenos Aires Argentina

**Keywords:** Complication, Orthopedic surgery, Clavien-Dindo, Healthcare quality

## Abstract

*Introduction*: Registration of adverse events following orthopedic surgery has a critical role in patient safety and has received increasing attention. The purpose of this study was to determine the prevalence and severity of postoperative complications in the department of orthopedic unit in a tertiary hospital.

*Methods*: A retrospective review from the postoperative complication registry of a cohort of consecutive patients operated in the department of orthopedic surgery from May 2015 to June 2016 was performed. Short-term complications (3 months after surgery), age gender, types of surgery (elective, scheduled urgency, non-scheduled urgency, and emergency), operative time, surgical start time (morning, afternoon or evening), American Society of Anesthesiologists score and surgeon's experience were assessed. Complications were classified based on their severity according to Dindo-Clavien system: Grade I complications do not require alterations in the postoperative course or additional treatment; Grade II complications require pharmacological treatment; Grade III require surgical, endoscopic, or radiological interventions without (IIIa) or with (IIIb) general anesthesia; Grade IV are life-threatening with single (IVa) or multi-organ (IVb) dysfunction(s), and require ICU management; and Grade V result in death of the patient. Complications were further classified in minor (Dindo I, II, IIIa) and major (Dindo IIIb, IVa, IVb and V), according to clinical severity.

*Results*: 1960 surgeries were performed. The overall 90-day complication rate was 12.7% (249/1960). Twenty-three complications (9.2 %) were type I, 159 (63.8%) type II, 9 (3.6%) type IIIa, 42 (16.8%) type IIIb, 7 (2.8%) type IVa and 9 (3.6%) were grade V according to Dindo-Clavien classification (DCC). The most frequent complication was anemia that required blood transfusion (27%) followed by wound infection (15.6%) and urinary tract infection (6%).

*Discussion*: The overall complication rate after orthopedic surgery in our department was 12.7%. The implementation of the DCC following orthopedic surgery was an important tool to measure the standard of care.

## Introduction

Postoperative complications are defined as any event that represents a deviation in the expected postoperative course and are associated with increased morbidity and mortality rate, hospital stay, decrease in quality of life and increase health costs [1]. Therefore, the prevalence of these complications can be used to measure the standard of care and quality of healthcare delivered to patient at a given institution. There is under-registration as well as a lack of a universally established classification system to track postoperative complications following orthopedic surgery [2]. Although diverse classification systems for orthopedic postoperative complications have been described, they are often highly complex and difficult to reproduce [3,4].

Due to the need of a standardized ranking system to classify surgical complication, the Dindo-Clavien classification (DCC) (Table 1), was originally described in General Surgery. This system contemplates a scale of 1–5 of increasing severity [5,6], and has shown good reproducibility in General Surgery [7]. It has been used in other subspecialties (Urology [8], Nephrology [9], and Gastroenterology [10]). Although it has been occasionally utilized to classify postoperative complications following hip and spine surgery [11,12], it has not been universally used in a register of postoperative complications after all the surgeries of an orthopedic unit.

**Table 1:** Classification of surgical complications according to Dindo-Clavien.

Grade	Definition
I	Any deviation from the normal postoperative course without the need for pharmacologic treatment or surgical, endoscopic and radiographic interventions
II	Requiring pharmacologic treatment with drugs Blood transfusions and total parenteral nutrition are also included
III	Requiring surgical, endoscopic, or radiographic intervention Intervention not/under (IIIA) under (IIIB) general anesthesia
IV	Life-threatening complication requiring IC/ICU management Single-organ dysfunction (IVA)/Multi-organ dysfunction (IVB)
V	Death of a patient

The purpose of this study is to determine the short-term postoperative complication rate of our unit, establishing the classification of Dindo-Clavien to standardize the registration and to evaluate the risk factors associated with major and minor complications.

## Materials and methods

A retrospective review of prospective collected data from the postoperative complication registry of 1984 consecutive patients operated in the department of orthopedic surgery at the *Hospital italiano de San Justo* (125 beds, 12 orthopedic surgeons, a nosocomial that signs a part of the healthcare network of the Hospital Italiano de Buenos Aires) from May 2015 to June 2016 was done. Twenty-four patients surgically treated and transferred from another center and patients previously operated in the same anatomical area (revision surgery) were excluded, leaving 1960 patients for analysis.

All patients were followed up to register any clinical or surgical complication within a 90-day postoperative period, with assessment at 15, 30 and 90 days after surgery (postoperative control). Data were recorded in a prospectively collected registry completed by staff, fellows and residents. In addition, two trained fellows performed a 90-day clinical chart revision. A complication was defined as any deviation from the normal postoperative state [1]. Two entities were defined and differentiated from the definition of complication: (1) sequelae, defined as any event expected as a result of surgery (e.g., limb amputation that leaves the inability to walk without assistance; and (2) treatment failure, defined as a phenomenon related to the pathology (e.g., recurrence of a tumor after oncological resection, or the requirement of a additional surgical debridement secondary to an abscess or osteomyelitis). Intraoperative complications were not considered in this study.

Complications were classified considering their severity according to DCC [5]: Grade I surgical complications that do not require alterations any additional pharmacological, surgical, endoscopic, or radiological interventions to treat it; Grade II complications require pharmacological treatment, including blood transfusions and total parenteral nutrition; Grade III complications require surgical, endoscopic, or radiological interventions; Grade IV complications are life-threatening single/multi-organ dysfunction(s) and require ICU management; and Grade V complications result in death of the patient (Table 1). Complications were further classified into minor (DCC I, II, IIIa) and major (DCC IIIb, IVa, IVb and V), according to clinical severity.

Data were collected on patient's age and gender. The type of surgery were classified as: *elective*, if the procedure was planned in advance and scheduled without time conditioning; *scheduled urgency* conditions that must be resolved within 48–72 h of the event to decrease clinical deterioration (e.g., closed femoral shaft fractures); *non-scheduled urgency* was defined as a condition that requires surgery within 6–12 h (e.g., open fractures, dislocations, and compartment syndrome); and *emergency* for those scenarios in which surgical intervention must be performed within the first 3 h due to life-threatening conditions (e.g., sepsis). Length of the procedure, time of starting (morning group: surgeries starting from 8 am to 11:59 am, afternoon group: from 12 pm to 5:59 pm, or evening group: from 6 pm to 7:59 am) were also considered. The American Society of Anesthesiologists (ASA) [13] and surgeońs experience (we considered experienced surgeons those with more than 6 years training) was also recorded. A full-time research fellow screened all patients who met the selection criteria. Baseline demographics, clinical history and complications were ascertained from electronic medical records.

### Ethics consideration

The study protocol (Protocol number 3220) was approved by the ethic review board from Hospital Italiano de Buenos Aires in concordance with Helsinki declaration.

### Statistical analysis

Descriptive analysis for continuous variables is shown as mean and standard deviation or median and interquartile range, according to the observed distribution. Categorical variables are expressed as absolute number and percentage. Comparisons between groups at baseline were performed with the chi-square test for categorical variables and the Mann–Whitney *U* test for continuous variables. Significance was defined as *p* < 0.05. All data analyses were performed with STATA software, version 13.

## Results

From 1960 surgeries analyzed, patients median age was 51.4 years (range 5–91), 1224 (62%) were female and 736 (38%) males. Overall, 249 complications were registered (12.7%) in 234 patients within the 3-month postoperative period. There were 13 patients that had more than one complication (5.5%). The median age of the complicated group was 68 years (IQR 53–79). Sixty-three percent of complications were in female patients (*n* = 147).

### Complications according to Dindop-Clavien classification (DCC)

Distribution of complications according to DDC were as follows: 23/249 (9.2%) complications were classified as type I, 159/249 (63.8%) type II, 9/249 (3.6%) type IIIa, 42/249 (16.8%) type IIIb, 7/249 (2.8%) type IVa and 9/249 (3.6%) were graded as type V, there were no patients with type IVb (Table 2). On the basis of severity, 64% (*n* = 191) were minor complications and 36% (*n* = 58) were major.

**Table 2:** Complications registered and DCC grade.

Complication	Number	%	DCC grade*
Anemia	67	27	II
Superficial wound infection	27	10.8	II
Urinary tract infection	16	6.4	II
Vomiting/diarrhea	15	6	I
Deep wound infection	12	4.8	IIIb
Loss of implant reduction	12	4.8	IIIb
Deep venous thrombosis	10	4	II
Death	9	3.6	V
Prosthesis dislocation	9	3.6	IIIa–b
Wound dehiscence	8	3.2	I–IIb
Acute urinary retention	8	3.2	II
Pneumonia	8	3.2	II
Wound hematoma	8	3.2	I–II
Symptomatic electrolyte abnormality	6	2.4	I
Excessive postoperative pain	5	2	I
Myocardial infarction	4	1.6	IVa
Atrial fibrillation	4	1.6	IVa
Respiratory failure	3	1.2	IVa
Periprosthetic fracture	3	1.2	IIIb
Postoperative paralysis	3	1.2	IIIb
Sudeck syndrome	2	0.8	II
Others	10	4	I–II

* **DCC grade**: Grade I (*n* = 23/9.2%), grade II (*n* = 159/63.8%), grade IIIa (*n* = 9/3.6%), grade IIIb (*n* = 42/16.8%), grade IVa (*n* = 7/2.8%), grade IVb (*n* = 0/0%) and grade V (*n* = 9/3.6%).

Within the 159 type II complications, the most frequent was symptomatic anemia 42.1% (67/159), followed by wound infection that required antibiotics 16.9% (27/159) urinary tract infection 10% (16/159) and deep venous thrombosis 6.2% (10/159).

Regarding complications that required an additional surgery (IIIb complications) the most common event was postoperative infection 28.6% (12/42) that required surgical debridement.

No significant differences were found in sex (*p* = 0.06), surgical start time (*p* = 0.33) and operative time (*p* = 0.75), ASA score (*p* = 0.117) or surgeon's experience (*p* = 0.44) and the severity of complications according to DCC. Regarding to age, we observed a direct relationship between age and higher grade of major complications with a median age of 58 (RIC 48–68) in grade 1 and 85 (68–92) in grade 5. The differences were significative (*p* = 0.0003).

### Surgery distribution and complication rate by orthopedic subspecialty

Table 3 shows surgeries distribution and complications based on subspecialty and DCC distribution.

**Table 3:** Patient distribution and orthopedic complication rate according to region and DCC grades.

Region	I	II	IIIa	IIIb	IVa	IVb	V	Total surgeries	Complication (*n*)	%
Knee surgery	1	43	2	9	3	0	0	445	58	13
Foot and ankle	3	21	2	4	0	0	0	304	30	9.8
Trauma	1	33	1	8	2	0	2	296	47	15.8
Upper extremity	2	3	1	4	0	0	0	241	10	4.1
Shoulder	7	7	0	2	0	0	0	171	16	9.3
Spine	6	16	1	5	0	0	2	163	30	18.4
Hip surgery	2	28	2	7	2	0	2	122	43	35.2
Pediatric	1	5	0	0	0	0	0	68	6	8.8
Oncology	0	1	0	0	0	0	0	28	1	3.5
Emergency room	0	2	0	3	0	0	3	122	8	6.5

### Surgical length and surgical start time

In the analysis of patients with complications, median surgical length was 75 min (IQR 60-120) there was no significant differences between minor and major complications (*p* = 0.9). No differences were observed in 1–5 Dindo-Clavien grades (*p* = 0.55). Regarding to surgical start time there were no differences between major and minor complications in the morning, afternoon and evening groups.

### Elective, urgent and emergent surgery

Among 249 complications, 58% (*n* = 145) occurred in elective surgeries, 40% (*n* = 101) in urgent surgeries and 2% (*n* = 3) in emergent surgeries. In elective surgeries 82% of complications were minor and 18% were classified as major, in scheduled urgencies the percentage is similar (78% minor and 22% major), on the other hand, non-scheduled urgency surgery had higher rate of major complications (48%), among the three complications observed in the emergency group, two were major (66%), the differences were significant (*p* = 0.001).

Table 4 shows comparison between type of surgery (elective, scheduled urgency, non-scheduled urgency and emergency) and DCC system. The most common complications reported in our institution were from knee and hip surgery: total knee arthroplasty (n = 52), followed by intertrochanteric hip fractures (n = 25) and medial hip fractures (n = 24).

**Table 4:** Major and minorcomplications and type of surgery.

	Major	%	Minor	%	Total
Elective	26	18	119	82	145
Scheduled urgency	18	22	64	78	82
Non-scheduled urgency	9	47	10	53	19
Emergency	2	66	1	33	3

Multivariate analysis was performed (Table 5), female sex resulted in increase risk for major compared with minor complications, on the other hand; electives and scheduled urgencies were associated with decreased risk of major complications.

**Table 5:** Multivariateanalysisfortherisk of majorcomplications.

	OR crude (IC 95%)	*p* value	OR adjusted (IC 95%)	*p* value
Female sex	2.37 (1.29–4.37)	0.005	2.34 (1.25–4.38)	0.008
Elective surgeries	0.24 (0.09–0.66)	0.005	0.24 (0.09–0.66)	0.006
Scheluled surgeries	0.31 (0.11–0.88)	0.029	0.34 (0.11–0.96)	0.041
Non-scheduled surgeries	2.22 (1.17–28.86)	0.0542	1.88 (1.37–25.64)	0.637
Surgical length	1 (0.99–1)	0.487	1 (0.99–1)	0.265

## Discussion

We observed an overall short-term complication rate of 12% in orthopedic surgery during our study period. Distribution of complications according to DDC were as follows: 23/249 (9.2%) complications were classified as type I, 159/249 (63.8%) type II, 9/249 (3.6%) type IIIa, 42/249 (16.8%) type IIIb, 7/249 (2.8%) type IVa and 9/249 (3.6%) were graded as type V, there were no patients with type IVb (Table 2). On the basis of severity, 64% (*n* = 191) were minor complications and 36% (*n* = 58) were major.

The report of postoperative complications in orthopedic surgery is an important tool to measure the standard of care of a medical institution; in addition, the prevalence of complications and the study of its causes can help the surgical team to formulate different interventions to reduce or avoid their occurrence, improving patient security and reducing healthcare costs. A systematic review of randomized controlled trials has shown that many orthopedic studies lack homogeneous and standardized reporting of complications, making the outcome comparison among different studies even more difficult [2]. This same principle applies to orthopedic surgery practice. Therefore, we decided to apply the system developed by Clavien [5] that is based on the main treatment implemented to manage a complication; since this score system has shown a reliable inter-observer reproducibility [5,7].

In general surgery, some authors adapted the DCC system to different subspecialties [8–10] with favorable results and acceptable interobserver reliability. They conclude that sub specialty adaptation provides an objective and more reproducible assessment able to be compared among institutions. In orthopedics; Sink et al. adapted the DCC to hip surgery and showed a high interobserver and intraobserver reliability when compared different clinical scenarios of hip preservation surgery [11]. Additionally implementation of DCC in spine surgery also resulted in an important tool to measure surgical outcomes [12]. However, to the best of the author's knowledge, the DCC has not been used in a department of orthopedic surgery to systematically record global postoperative complications.

The main complication observed was symptomatic anemia that required blood transfusion followed by wound infection requiring antibiotics or surgical debridement.

The most common procedures related to any complication were total knee arthroplasty (TKA), intertrochanteric hip fractures that required open reduction and internal fixation (ORIF) with endomedular device and cervical hip fractures that required total hip arthroplasty, similar results were observed by Molina et al. [14].

Postoperative transfusions in orthopedic surgery vary significantly among clinicians and hospitals [15] and even when there are protocols to perform a blood transfusion they are not consistently used [16]. A 2013 study of 5.820 knee and hip arthroplasty surgeries found differing transfusion rates: 4.8%–63.8% for total knee and 4.3%–86.8% for THA [17]. The decision criteria for blood transfusion is not a static value, the clinical status of the patient should be taken into account [18], however, one of the most important factors considered is the preoperative hemoglobin level [19] and was the main factor considered in our institution.

When analyzing the group of 249 patients that had a 90-day postoperative complications, we observed an association between increased age and high-grade complications (DCC IIIb, IV, V) compared to minor (DCC I, II, IIIa) with significant differences. Similar results were observed in other studies [20,21]. Molina et al [14], on the other hand, observed an increase rate of minor complications in patients more than 65 years.

Prolonged operative time is known to increase the risk of major postoperative complications [22,23]. However, duration of surgery alone could be not a major determinant of complications and other factors should be considered [24]. In our study, we found no association between operative time and higher grade of complications; those results can be explained due to the heterogeneous group of surgeries performed.

Regarding to the analysis of type of surgery, we found higher grade of major complications in non-scheduled urgency and emergency surgeries compared with elective and scheduled urgencies.

This study is not free of limitations; firstly, the evaluation of a heterogeneous group of surgeries, ranging from ambulatory procedures to complex surgeries makes the comparison more difficult. The variation between different orthopedic regions can be explained by several factors such as complexity or ambulatory status. The lowest rate of complications was observed in oncologic procedures, which are mainly ambulatory soft tissue resections in contrast with knee or trauma surgery, which had higher complication rate. In addition, a 90-day observation period is relatively short, making the registry of long-term complications such as late postoperative infection, periprosthetic fractures after 3 months or non-union not possible.

Probably a further analysis of complication based on sub-specialties should be performed to analyze the usefulness of DCC. However, the proper registry using a uniform classification system allows understanding some outcomes, in addition, the effort to use a simple system of reporting complications can be helpful to analyze and compare postoperative results among different institutions.

## Conclusion

The overall complication rate after orthopedic surgery of our unit was 12.7% and 2.8% were classified as major complications, which were more common in females, non-scheduled surgeries and emergency surgery when compared to minor complications. DCC system was useful for the purpose of our study, however, further adaptations based on type of surgery or subspecialty could improve the usefulness of this classification. The report of postoperative complications is an important tool to measure the standard of care.

## Conflict of interest

The authors declare that they have no conflict of interest in relation with this paper.
